# Venom Concentrations and Clotting Factor Levels in a Prospective Cohort of Russell’s Viper Bites with Coagulopathy

**DOI:** 10.1371/journal.pntd.0003968

**Published:** 2015-08-21

**Authors:** Geoffrey K. Isbister, Kalana Maduwage, Fiona E. Scorgie, Seyed Shahmy, Fahim Mohamed, Chandana Abeysinghe, Harendra Karunathilake, Margaret A. O’Leary, Christeine A. Gnanathasan, Lisa F. Lincz

**Affiliations:** 1 School of Medicine and Public Health, University of Newcastle, Callaghan, New South Wales, Australia; 2 Department of Clinical Toxicology and Pharmacology, Calvary Mater Newcastle, Newcastle, New South Wales, Australia; 3 South Asian Clinical Toxicology Research Collaboration (SACTRC), Faculty of Medicine, University of Peradeniya, Peradeniya, Sri Lanka; 4 Department of Biochemistry, Faculty of Medicine, University of Peradeniya, Peradeniya, Sri Lanka; 5 Hunter Haematology Research Group, Calvary Mater Newcastle, Newcastle, New South Wales, Australia; 6 District Hospital Hingurakgoda, Hingurakgoda, Sri Lanka; 7 General Hospital Matara, Matara, Sri Lanka; 8 Department of Clinical Medicine, Faculty of Medicine, University of Colombo, Colombo, Sri Lanka; Instituto Butantan, BRAZIL

## Abstract

**Background:**

Russell’s viper envenoming is a major problem in South Asia and causes venom induced consumption coagulopathy. This study aimed to investigate the kinetics and dynamics of venom and clotting function in Russell’s viper envenoming.

**Methodology/Principal Findings:**

In a prospective cohort of 146 patients with Russell’s viper envenoming, we measured venom concentrations, international normalised ratio [INR], prothrombin time (PT), activated partial thromboplastin time (aPTT), coagulation factors I, II, V, VII, VIII, IX and X, and von Willebrand factor antigen. The median age was 39y (16–82y) and 111 were male. The median peak INR was 6.8 (interquartile range[IQR]:3.7 to >13), associated with low fibrinogen [median,<0.01g/L;IQR:<0.01–0.9g/L), low factor V levels [median,<5%;IQR:<5–4%], low factor VIII levels [median,40%;IQR:12–79%] and low factor X levels [median,48%;IQR:29–67%]. There were smaller reductions in factors II, IX and VII over time. All factors recovered over 48h post-antivenom. The median INR remained >3 at 6h post-antivenom but had reduced to <2, by 24h. The aPTT had also returned to close to normal (<50sec) at 24h. Factor VII, VIII and IX levels were unusually high pre-antivenom, median peak concentrations of 393%, 307% and 468% respectively. Pre-antivenom venom concentrations and the INR (r = 0.20, p = 0.02) and aPTT (r = 0.19, p = 0.03) were correlated (non-parametric Spearman analysis).

**Conclusions:**

Russell’s viper coagulopathy results in prolonged aPTT, INR, low fibrinogen, factors V, VIII and X which recover over 48h. Severity of clotting abnormalities was associated with venom concentrations.

## Author Summary

Snake envenoming is an important health issue in many parts of the world and coagulopathy is one of the commonest manifestations. Russell’s viper envenoming occurs throughout south-east and south Asia and most commonly causes a venom induced consumption coagulopathy which may be complicated by bleeding. There is a limited understanding of the time course of the coagulopathy, including the specific clotting factor deficiencies and their relationship to the time course of venom concentrations. In this study, we measure the venom concentrations and clotting factor concentrations throughout the course of the patient admission. The study confirms that Russell’s viper envenoming causes prolonged clotting times and deficiencies of factors V, X, VIII and fibrinogen which recover over 2 days post-antivenom. In addition, the prothrombin time and activated partial thromboplastin times correlated with venom concentrations. Factor VII, VIII and IX levels were very high and may be related to venom activity, although further research is required.

## Introduction

Snake envenoming is a major health issue in the Asia-Pacific region with between 250,000 and 1 million cases occurring annually.[1] Russell’s viper (*Daboia russelii*) is one of the most medically important snakes in the region,[1, 2] with bites causing death in 2 to 5% of cases, accounting for the majority of fatal snakebites in Sri Lanka[3]. Russell’s viper envenoming results in local effects, venom induced consumption coagulopathy (VICC), mild neurotoxicity and renal injury.[3, 4] VICC is the commonest systemic manifestation and in some cases results in mucosal bleedings and less commonly major bleeding including intracranial haemorrhage.[3, 5–7]

The *in vitro* procoagulant effects of Russell’s viper venom have been well characterised and a number of procoagulant toxins have been isolated and used in laboratory assays for decades.[8–10] Russell’s viper venom contains both factor X and factor V activators which trigger the clotting pathway early on, resulting in consumption of multiple clotting factors.[11–13] Previous studies have shown that VICC resulting from Russell’s viper envenoming causes overall haemostatic disturbances which manifest in prolonged prothrombin time (PT)/international normalised ratio (INR) and activated partial thromboplastin time (aPTT), as well as decreased levels of fibrinogen, factor V and factor X and elevated D-Dimer concentrations.[14–17] However, there is limited information on the dynamics of clotting factor levels in Russell’s viper envenoming and their response to antivenom treatment.

A number of studies have measured venom concentrations in patients with Russell’s viper envenoming, and some have suggested that there is recurrence of venom post-antivenom.[5, 18] However, there is limited information on the dynamic relationship between venom concentrations and clotting factor levels, and whether the detection of venom post-antivenom is associated with further clotting factor consumption.

This study aimed to explore the dynamic changes in venom and clotting factor levels in VICC, including the effect of antivenom treatment following Russell’s viper envenoming.

## Methods

This was a prospective observational study of serial venom concentrations and clotting factor levels in definite Russell’s viper (*D*. *russelii*) envenomed patients admitted to a single hospital in Sri Lanka. Patients were recruited as part of a large cohort study of snakebites admitted to the Base Hospital Chilaw in Central West Sri Lanka[19]. The study was approved by the Ethical Review Committee, Faculty of Medicine, University of Colombo, Sri Lanka. All patients gave written and informed consent for the collection of clinical data and blood samples.

### Patients

All patients (>13 years old) admitted with a suspected or definite snake bite between January 2007 and July 2009 were identified on admission to hospital. From these only definite cases were included if Russell’s viper venom was detected with a venom-specific enzyme immunoassay (EIA). All admission samples were tested for Russell’s viper venom. VICC was defined as coagulopathy (abnormal PT/INR) with evidence of consumption (low/undetectable fibrinogen or elevated D-Dimer more than 10 times the upper limit of normal) and an INR > 1.5.[20] Severe or complete VICC was defined as an INR > 13 (unrecordable).

### Data collection

Baseline data, including demographic data (age and sex), information on the snake bite (snake type, time of bite), clinical effects (local effects: local pain, swelling, bruising, blistering and necrosis; systemic effects: features of coagulopathy including bleeding and neurotoxicity) and antivenom treatment (dose and time of administration) were recorded prospectively for all patients. Research blood samples were collected from all patients on admission and then at regular time intervals during their admission. Blood was collected in citrated tubes for clotting times and coagulation studies and in serum tubes for venom-specific EIA. All samples were immediately centrifuged, aliquoted and frozen initially at -20°C, and then transferred to a -80°C freezer within 2 weeks of collection. All patients received Indian polyvalent snake antivenom manufactured by VINS Bioproducts Limited (batch number: ASV 42C/06, 1030) or BHARAT Serum and Vaccines Limited, India (batch number: 5346KD4, LY 55/05, LY 32/04, A5307035).

### Venom-specific enzyme immunoassays (EIA)

A sandwich EIA was used to measure Russell’s viper venom in serum samples and has previously been described.[7, 21, 22] In brief, polyclonal IgG antibodies were raised against Russell’s viper (*D*. *russelii*) venom in rabbits[23]. Antibodies were bound to microplates as well as being conjugated to biotin for a sandwich EIA with the detecting agent being streptavidin-horseradish peroxidase. All samples were measured in triplicate, and the averaged absorbance converted to a concentration by comparison with a standard curve based on serial dilutions of venom using a sigmoidal curve. The assay does not cross-react with *Hypnale* venom, the only other medically important snake in Sri Lanka that cause coagulopathy (excepting *Echis carinatus—*saw-scaled viper—in the north). [22]

### Clotting studies and clotting factor assays

Frozen citrated plasma samples were used for all clotting times and clotting factor studies including prothrombin time (PT)/international normalised ratio [INR] and activated partial thromboplastin time (aPTT). Levels of factors I (fibrinogen), II (prothrombin), V, VII, VIII, IX and X, von Willebrand factor antigen (VWF:Ag) and D-Dimer were all measured. All assays were done using either standard coagulometric or immunoturbidimetric methods as provided by the manufacturer and were performed on a Behring Coagulation System (BCS) or Sysmex CA-1500 analyzer (Dade Behring, Marburg, Germany) [24]. Individual clotting factor levels were determined by mixing patient plasma with plasma deficient in the factor being measured and the time for clot formation measured in seconds. The amount of factor present in the sample was quantified by comparing with a standard or reference curve produced using serial dilutions of plasma deficient in the factor mixed with normal plasma, against the clotting time. The quantification of von Willebrand factor antigen (VWF:Ag) and D-Dimer was done using immunoturbidometric methods.

### Data analysis

The average number of samples collected from the patients was four (range 1 to 10). To describe the peak effect of the venom on the clotting pathway, the maximal (longest) clotting time or minimum (lowest) factor level was determined for each patient. Factor levels and clotting times were then reported as medians and interquartile ranges of the maximum (PT/INR, aPTT, D-Dimer) or minimum (Fibrinogen, Factors II, V, VIII, IX, X) for each level over the time course of the admission.

For visual analysis of concentration time data, median factor concentrations were plotted versus time to provide empirical estimates of the average/median changes over time in the coagulation studies and the factor levels. Time zero was defined as the time of antivenom administration. This was done by binning the data based on the time post-snake antivenom and then calculating the median factor level/clotting time for each bin and the median time for each bin (ie. for the specified time period). The median factor level/clotting time was then plotted versus the median time. For factors VII, VIII and IX, the median and interquartile range of the levels of these factors was taken from the bin where the peak occurred because of the unusually high pre-antivenom levels of these three factors. Separate plots were also made for complete VICC where the INR was unrecordable (INR > 13) and partial VICC where the INR was abnormal but still recordable (1.5 < INR < 13).

To investigate whether there was an association between the venom load (i.e. amount of venom delivered by the snake) and the severity of VICC, correlations between pre-venom concentrations and the PT/INR, aPTT and all clotting factor levels were tested with non-parametric Spearman correlation analysis. In addition clotting tests and factor studies versus pre-antivenom venom concentrations were plotted (with the line of best fit and 95% confidence intervals).

All analyses and graphics were done in GraphPad Prism version 6.01 for Windows, GraphPad Software, San Diego California USA, www.graphpad.com.

## Results

There were 146 patients with Russell’s viper bites. The median age was 39 years (Range: 16 to 82 years) and 111 (76%) were male. All patients had VICC (low fibrinogen and elevated PT) and 70 (48%) had neurotoxicity. Local effects were reported in 134 (91%) patients and systemic bleeding developed in 14 (10%) patients. The median pre-antivenom venom concentration was 201ng/ml (IQR: 74 to 435ng/ml; Range: 1 to 1521ng/ml) which dropped to a median concentration of 2ng/ml (IQR: 0 to 9ng/ml) after the administration of antivenom.

**Fig 1:**
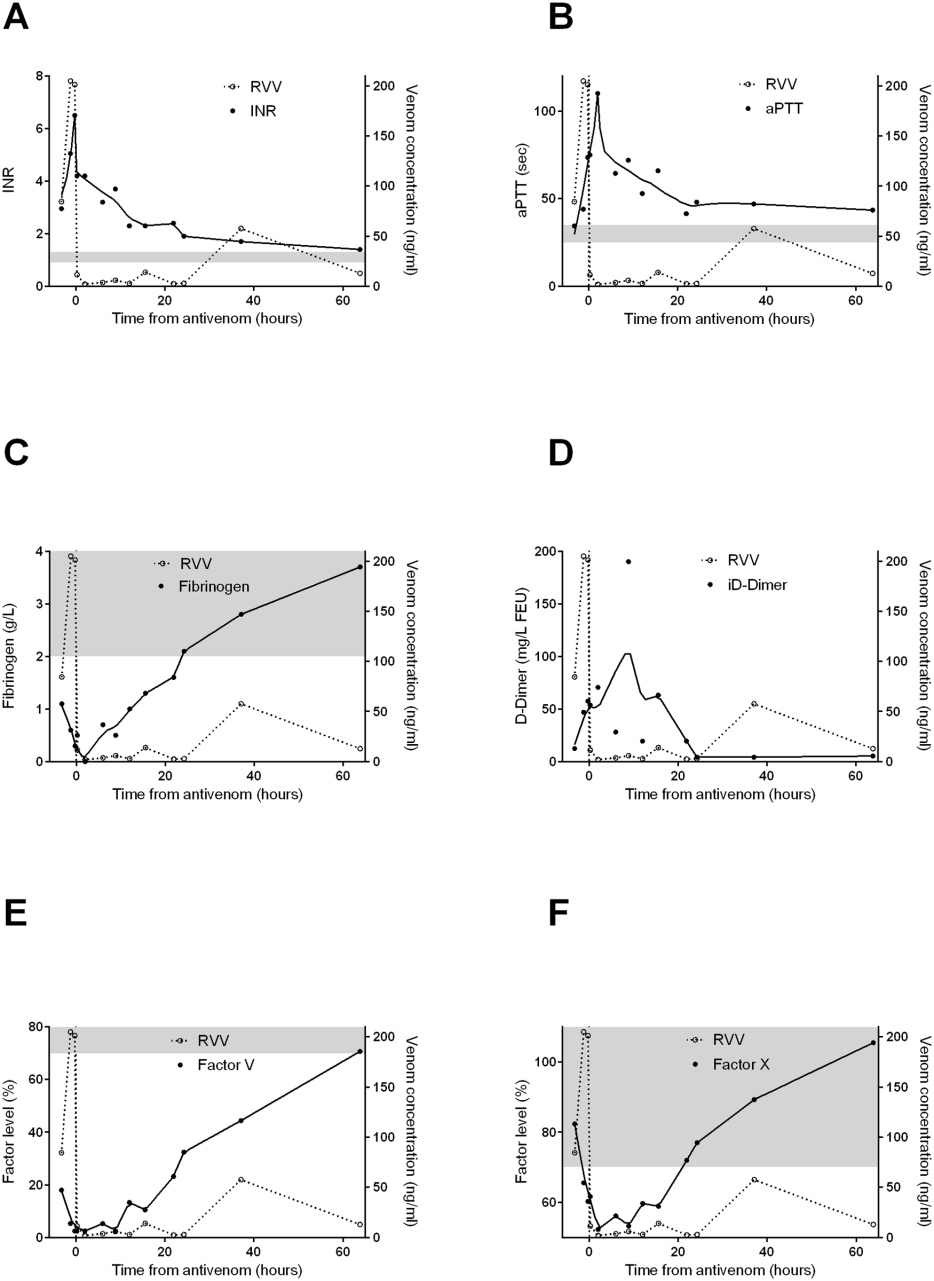
Plots of median venom concentrations (open circles [○] and dashed lines; all panels), clotting times and factor concentrations (filled circles [●]) versus time post-antivenom for 146 patients with Russell’s viper envenoming including INR [A], aPTT (sec) [B], fibrinogen (g/L) [C], iD-dimer (mg/L FEU) [D], factor V (%) [E] and factor X (%) [F]. Black lines represent the interpolated median factor concentration time curves and the shaded area is the normal range for each test. INR—international normalised ratio; aPTT—activated partial thromboplastin time; RVV—Russell’s viper venom.

**Fig 2:**
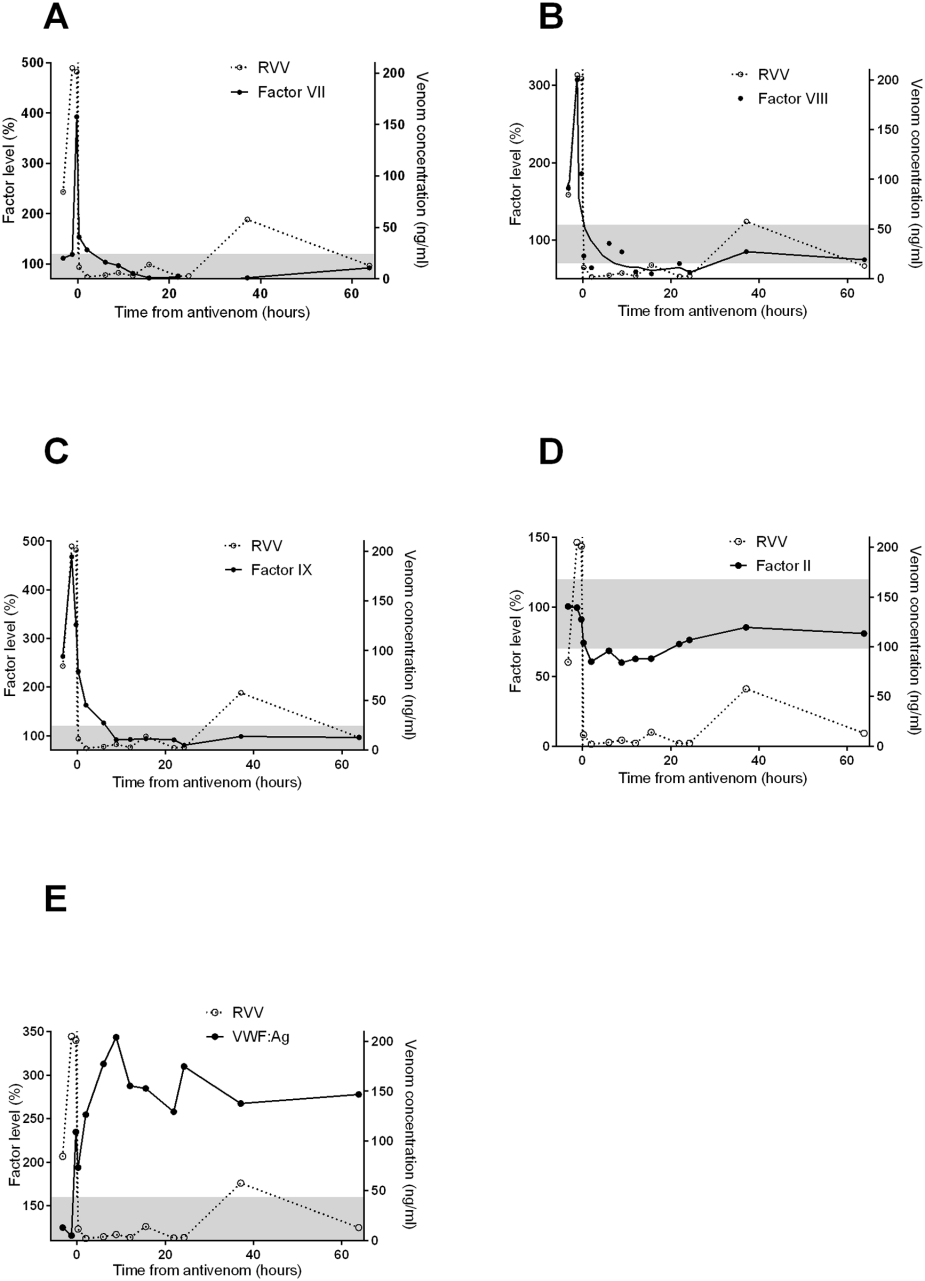
Plots of median venom concentrations (open circles [○] and dashed lines; all panels), clotting times and factor concentrations (filled circles [●] and lines) versus time post-antivenom for 146 patients with Russell’s viper envenoming including factor VII (A; thick lines), factor VIII (B), factor IX (C), factor II (D) and v-WF antigen (E). The black lines represent the interpolated median factor concentration time curves and the shaded area is the normal range for each test. RVV—Russell’s viper venom; VWF:Ag—von Willebrand factor antigen.

The median peak INR in the patients was elevated at 6.8 (IQR: 3.7 to >13) as was the median peak aPTT of >180sec (IQR: 91.3 to > 180sec). The abnormal clotting times were associated with a low fibrinogen [median, <0.01g/L; IQR: <0.01 to 0.9g/L], low factor V levels [median <5%; IQR: <5 to 4%], low factor VIII levels [median; 24%; IQR: 10 to 41%] and mildly decreased factor X levels [median 48%; IQR: 29 to 67%] over the course of the patient admissions. The median of the highest or median of the lowest factor concentrations/clotting times are given in Table 1. There were smaller reductions in factors II, VII and IX over the course of the patient admission. The INR, fibrinogen, factors V and X recovered over 48 hours post-antivenom (Figs 1 and 2). The median INR remained greater than 3 at 6 hours post-antivenom, but had reduced to less than 2, by 24 hours (Fig 1). The aPTT had also returned to close to normal (< 50sec) at 24 hours (Fig 1). There were smaller reductions in most factor levels in partial versus complete VICC (S1 Fig).

**Table 1:** The median, interquartile range (IQR) and range of the minimum (Factors I, II, V, VII, VIII, IX, X) or maximum (PT/INR, aPTT, D-Dimer) factor concentrations/clotting times measured for the 146 patients during their hospital admission.

Factor Concentration or Clotting times	Normal Range	Median	Interquartile range	Range
**Prothrombin time (PT) sec**	9–14	69	36–180	12–180
**INR**	0.9–1.3	6.8	3.7 –>13	1.3 –>13
**aPTT (s)**	25–35	> 180	91.3 –> 180	29 –> 180
**Fibrinogen (g/L)**	2–4	< 0.01	<0.01–0.9	<0.01–3
**Factor II (%)**	70–120	60	49–74	10–120
**Factor V (%)**	70–120	< 5	<5–4	<5–61
**Factor VII (%)**	70–120	63	43–123	15–1203
**Factor VIII (%)**	70–120	24	10–41	1–335
**Factor IX (%)**	70–120	88	66–109	2–860
**Factor X (%)**	70–120	48	29–67	<0.01–152
**VWF:Ag (%)**	50–160	176	100–245	39–523
**D-dimer (mg/L)**	< 0.5	134	20–450	1–905

Factors VII, VIII and IX levels were very high prior to antivenom and then dropped dramatically into the normal range or to low levels (FVIII) post-antivenom (Fig 2). The median peak factor VII levels were 393% (IQR: 85 to 698%), factor VIII levels were 307% (IQR: 160 to 400%) and factor IX levels were 468% (IQR: 331 to 704%) respectively (Fig 2).

There was a statistical association between pre-antivenom venom concentrations and the INR (r = 0.20, p = 0.02), aPTT (r = 0.19, p = 0.03) and factor IX (r = -0.36, p<0.001), and there were trends for factor V (r = -0.17, p = 0.058), factor X (r = -0.17, p = 0.05) and VWF:Ag (r = -0.18, p = 0.053) (S2 Fig). There were no statistical associations for pre-antivenom venom concentrations and fibrinogen, factors II, VII, VIII and D-Dimer (S2 Fig).

## Discussion

The study shows that VICC resulting from Russell’s viper envenoming is characterised by an elevated INR and aPTT associated with low fibrinogen, factor V, VIII and X levels. There was an association between the pre-antivenom venom concentrations and the severity of the coagulopathy, mainly with the INR and aPTT. The coagulopathy resolved over a period of 48 hours after the administration of antivenom consistent with VICC from other snakes.[24, 25] An unusual finding was the very high levels (above the normal range) of factor VII, VIII and IX prior to antivenom treatment which then returned to normal ranges soon after antivenom treatment. It is unclear the exact reason for these high factor levels but may be related to venom activity in the sample *in vitro*.

**Fig 3:**
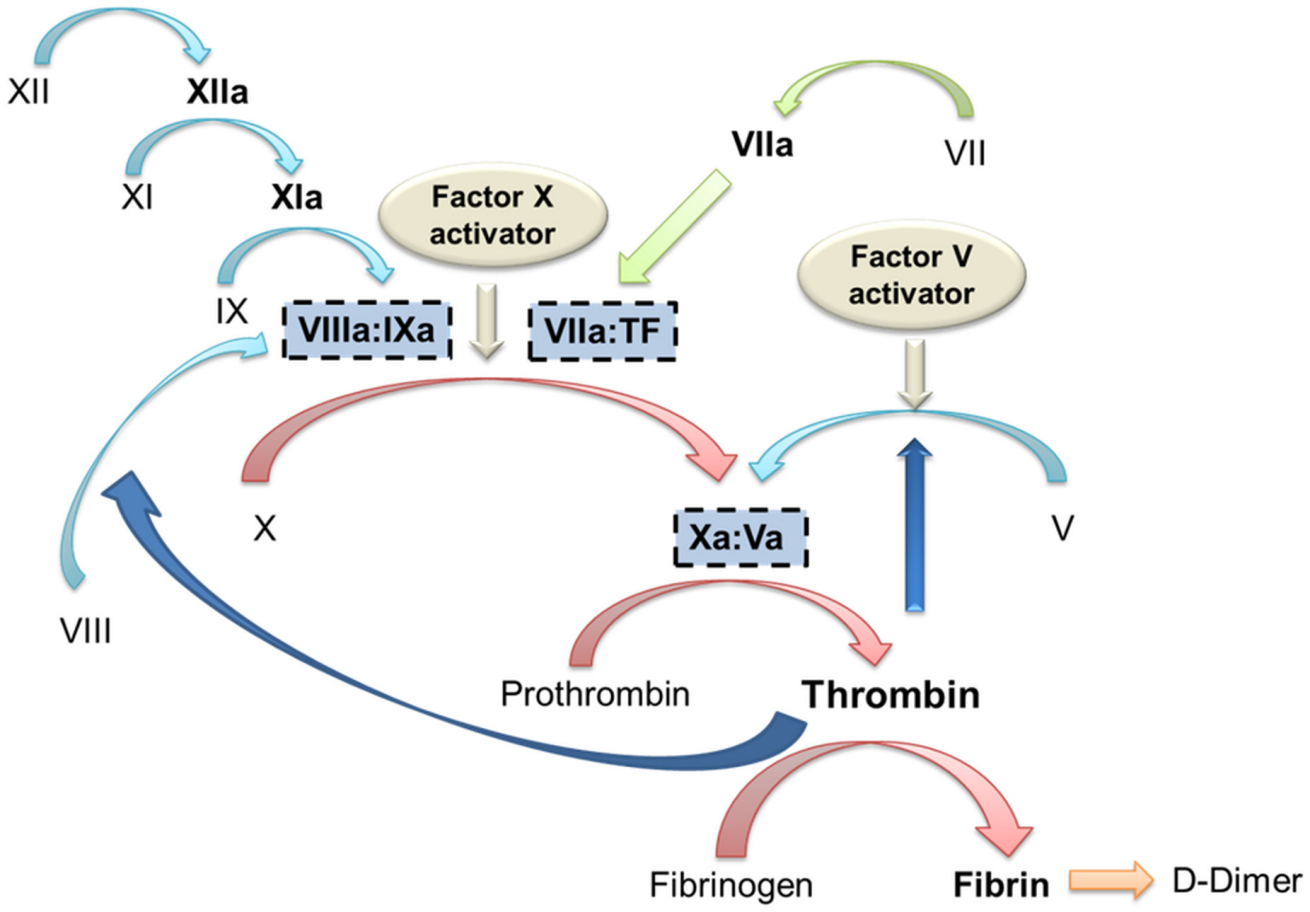
Diagrammatic representation of the clotting pathway and the points where Russell’s viper factor X and factor V activators cause activation of the clotting pathway.

Russell’s viper venom contains factor V and factor X activators which convert these factors to their activated forms (i.e. Va and Xa), explaining the low factor V and factor X levels in human envenoming (Fig 3).[11, 12, 26, 27] Activation of factor V and X results in the formation of the prothrombinase complex (XaVa) which activates the whole clotting cascade by converting prothrombin to thrombin. This then leads to the consumption of fibrinogen, factor VIII and further consumption of factor V. The multiple factor deficiencies result in the prolonged INR and aPTT, and activation of the clotting cascade leads to increased fibrinolysis and therefore elevated D-Dimer levels. A similar pattern of factor deficiencies has been described in previous studies of patients envenomed by Russell’s vipers.[14–17] Four studies showed the initial drop in fibrinogen to low levels followed by a recovery over 24 to 48 hours. [14–17] Two studies also reported serial measurements of factor V and factor X, with initial low levels that recovered over 24 hours consistent with our study.[15, 16]

The recovery of the coagulopathy occurred over a period of 48 hours, although the rate of recovery differed for each of fibrinogen, factor V and factor VIII. The median INR was still greater than 3 at 6 hours, suggesting that 6 hours if too early to determine the effect of antivenom (Fig 1). The median INR was 2.3 at 12 hours and then less than 2 at 24 hours suggesting that patients had only a mild coagulopathy at this time (Fig 1).

The presence of very higher factor VII, VIII and IX levels (392%, 313% and 463% respectively; Fig 2) prior to antivenom is an unexpected finding, although earlier studies have reported high values in a small number of patients.[15, 16] A possible explanation for this finding is that the presence of Russell’s viper venom (RVV) factor X activator toxin in the sample results in falsely high factor levels. The *in vitro* activity of the toxin would appear similar to the activity of factor VII, VIII and IX. For example, in the case of factor VII, the presence of active RVV in the sample will result in a shorter clotting time because the RVV factor X activator has the same activity as factor VII (i.e. both VII:TF and RVV factor X activator convert factor X to Xa). Interpolation of this shorter clotting time on the standard curve for factor VII results in a factor VII level greater than 100% (S3 Fig). A similar phenomena occurs with both factor VIII and factor IX because the VIIIa:IXa complex also activates factor X to Xa (intrinsic pathway). Previous studies that have measured factor VII, VIII and IX levels have also found normal to high values, but because they have only measured factors at one time point, they are difficult to interpret.[14, 16] The time course of these three factors, with high levels only occurring in the pre-antivenom samples, and then normal factor VII levels or low factor VIII and IX levels post-antivenom, also suggests that these high levels prior to antivenom are due to the assay and not VICC (Fig 2).

There was only moderate reduction in factor II, and moderate reductions in factors VII and IX after the initial high pre-antivenom values of the latter two. This is most likely because none of these factors are directly activated by the venom (as are factors V and X), or are factors that are completely consumed when the clotting pathway is completely activated (factor VIII). There is a large excess of factor II (prothrombin), so even if all the fibrinogen is converted to fibrin (consumed), prothrombin levels will only partially decrease. Factor VII and IX are also not activated and consumed in VICC. VWF:Ag levels were mildly elevated which is most likely an indirect consequence of the clotting pathway being activated.

There was a rebound in venom concentrations approximately 40 hours after antivenom (Figs 1 and 2) which we have previously shown to not be associated with recurrent coagulopathy.[28] A recent study has shown that this re-appearance of venom is due to bound venom being detected by the venom specific EIA.[29] This is entirely consistent with the results in this study. There was recovery of all clotting factors and clotting times despite this rebound in measured venom This further supports that venom-specific EIA is measuring bound venom post-antivenom.

The study found a correlation between venom concentrations and the severity of the coagulopathy measured by the INR and aPTT. An earlier study by Than et al found a similar association between venom concentrations and the severity of the coagulopathy.[15] There was an association between venom concentrations and factor V and Factor X, consistent with the Factor V activator and Factor X activator toxins in the venom.

There are a number of limitations of the study. Coagulation studies are best done on samples collected fresh from the patient. This was not possible in this study because of the resource limitations in the hospital where the patients were treated. However, all samples were immediately centrifuged, aliquoted and frozen to preserve the functional activity of the clotting factors. The results were in keeping with another study of VICC in Australian elapids which used a similar approach successfully.[24]Platelet counts, platelet function testing and tissue factor assays were not done in this study. These may play a role in haemostatic disturbances, particularly platelets, and should be investigated in future studies.

Another limitation was that the decision to give antivenom was made by the treating clinician and not the investigators. However, the majority of these patients were recruited to a randomised controlled trial comparing two different infusion rates of antivenom. This meant that all patients had a standardised antivenom administration.

## Supporting Information

S1 Fig Plots of median venom concentrations (red circles and lines; all panels), clotting times and factor concentrations (blue circles and lines) versus time post-antivenom for 51 patients with complete VICC (closed circles [●] and lines; column 1) and 94 with partial VICC (open circles [○] and dashed lines; column 2), including INR [A], fibrinogen (g/L) [B], iD-dimer (mg/L FEU) [C], factor V (%) [D] and factor X (%) [E]. The shaded area is the normal range for each test. INR—international normalised ratio; RVV—Russell’s viper venom.
(TIF)

S2 Fig Plots of clotting tests and factor levels versus pre-antivenom (AV) venom concentrations showing the linear regression with 95% confidence intervals for international normalised ratio (INR; A), activated partial thromboplastin time (aPTT; B), factor V (C), factor X (D), factor VIII (E), factor IX (F), v-WF antigen (G) and fibrinogen (H) a. VWF:Ag—von Willebrand factor antigen. (TIF)

S3 Fig Diagram of the steps in the factor VII assay and the standard curve that is used to convert clotting times measured in the assay to factor VII concentrations. The standard curve is made by diluting normal plasma with factor VII deficient plasma and measuring a range of clotting times for decreasing dilutions of factor VII concentrations. In a normal sample where there is normal factor VII concentrations the measured clotting time will be approximately 12 seconds which corresponds to 100% factor VII concentration. Factor deficient patient samples will have a longer clotting time and therefore lower factor concentrations. However, when factor X activator from Russell’s viper venom (RVV) is present in the sample this will result in a shorter clotting time because RVV factor X activator has the same action as factor VII. In the diagram the clotting time is 2 seconds which is interpolated as 400% factor VII concentration. In this way the factor assay is a surrogate measure for the toxin activity.
(TIF)
