# Supplementary figures and images for: Venom Concentrations and Clotting Factor Levels in a Prospective Cohort of Russell’s Viper Bites with Coagulopathy
Source: PLoS Negl Trop Dis. 2015 Aug 21;9(8):e0003968. doi: 10.1371/journal.pntd.0003968 (PMC4546603; doi:10.1371/journal.pntd.0003968)

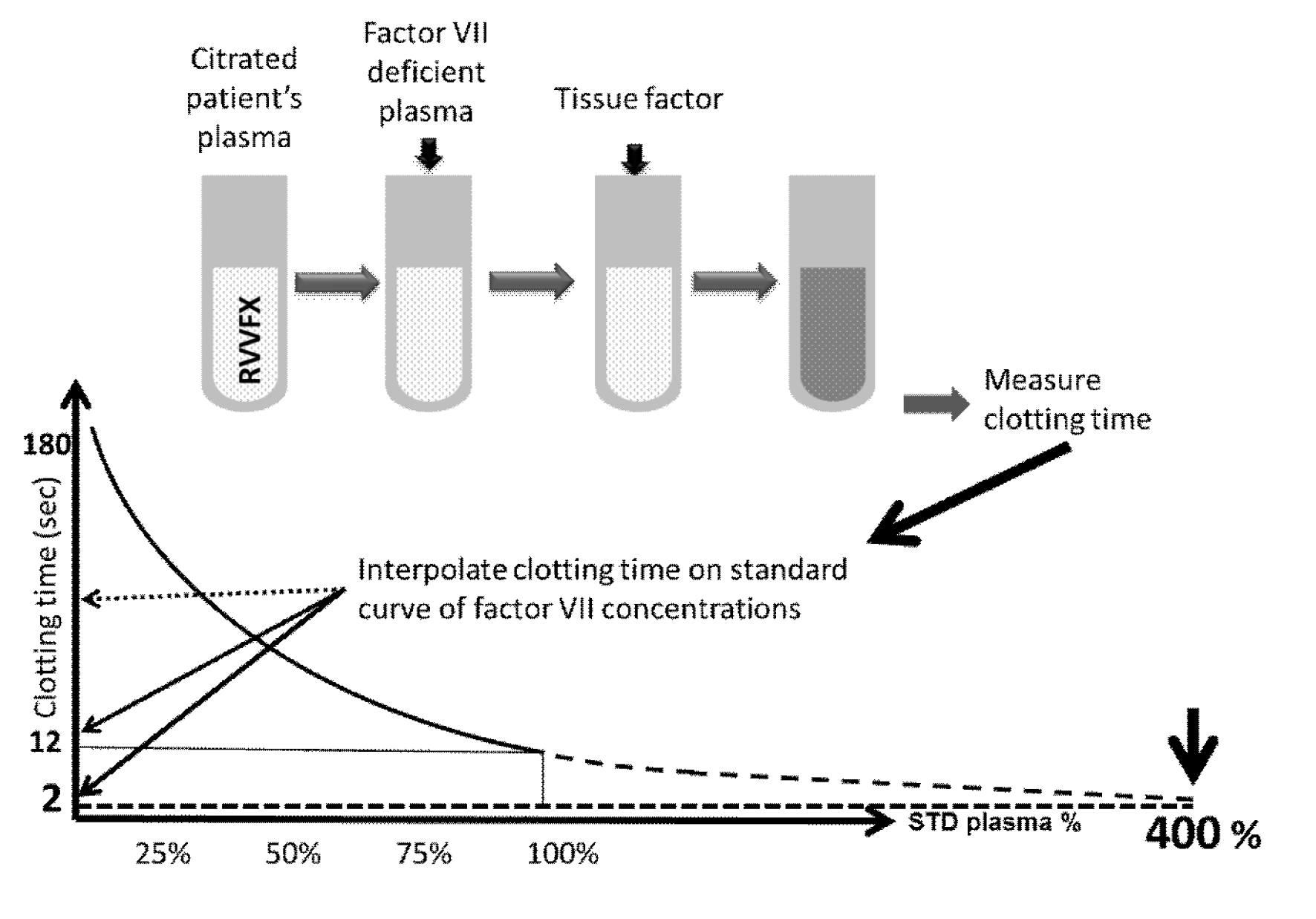

Supplement: S3 Fig — The standard curve is made by diluting normal plasma with factor VII deficient plasma and measuring a range of clotting times for decreasing dilutions of factor VII concentrations. In a normal sample where there is normal factor VII concentrations the measured clotting time will be approximately 12 seconds which corresponds to 100% factor VII concentration. Factor deficient patient samples will have a longer clotting time and therefore lower factor concentrations. However, when factor X activator from Russell’s viper venom (RVV) is present in the sample this will result in a shorter clotting time because RVV factor X activator has the same action as factor VII. In the diagram the clotting time is 2 seconds which is interpolated as 400% factor VII concentration. In this way the factor assay is a surrogate measure for the toxin activity. (TIF) [file pntd.0003968.s003.tif]
